# Fat’s all, folks: culturing and manipulating peri-prostatic adipocytes to probe impacts on prostate cancer biology

**DOI:** 10.1530/JOE-25-0256

**Published:** 2026-01-23

**Authors:** Nil Grunberg, Mathias Winkler, Giles Hellawell, Bijan Khoubehi, Taimur T Shah, Hashim Ahmed, Charlotte L Bevan, Claire E Fletcher

**Affiliations:** ^1^ Imperial Centre for Translational and Experimental Medicine, Division of Cancer, Department of Surgery and Cancer, Faculty of Medicine, Imperial College London, London, UK; ^2^ Imperial Prostate, Division of Surgery, Department of Surgery and Cancer, Faculty of Medicine, Imperial College London, London, UK; ^3^ Department of Urology, Imperial College Healthcare National Health Service (NHS) Trust, London, UK; ^4^ Department of Urology, Chelsea & Westminster NHS Foundation Trust, London, UK

**Keywords:** adipose, prostate cancer, explant, pre-adipocyte, adipocyte, prostate

## Abstract

Obesity, officially recognised as a global epidemic by the World Health Organization, will soon overtake smoking as the largest preventable risk factor for cancer. By 2035, more than half the world’s population is expected to be overweight or obese with a significant increase in obesity-related health expenditures. However, despite the increase in prevalence and the overall lower life expectancy associated with obesity, mechanisms underpinning obesity-driven diseases are not well understood. Adipocytes pose many challenges for *in vitro* culture due to their poor cell-to-surface attachment and low viability. Their large size and high lipid content can also present methodological challenges for downstream experiments. Several mouse and human-derived primary pre-adipocyte cell lines have been established over the years. However, they show limited renewal capacity and they cannot be cultured long term *in vitro*. Commercial cell lines available, which can be cultured long term, fail to represent organ-specific adipocyte heterogeneity. Adipose tissue from different organs and fat depots can show significant heterogeneity in terms of metabolism and overall secretome and extracellular matrix production. The prostate, for example, is surrounded by peri-prostatic adipose tissue (PPAT), the volume of which is associated with an increased risk of lethal prostate cancer and a reduced therapy response. Here, we outline a protocol for *ex vivo* culture of fresh PPAT and non-prostatic adipose tissue (NPAT), which reflects donor- and depot-specific characteristics. *Ex vivo* culture of PPAT/NPAT explants maintains cell–cell interactions and preserves local tissue architecture within adipose tissue. We have also described establishment of immortalised, patient PPAT-derived pre-adipocytes and patient-matched NPAT pre-adipocytes that can be *in vitro* differentiated into mature adipocytes. The protocols outlined here could be readily adapted to other organ-specific fat depots, such as mammary/bone marrow adipose tissue, and to tissues of non-human origin.

## Introduction

Obesity, termed a global epidemic by the World Health Organisation (WHO), is a complex, chronic disease, which will shortly overtake smoking as the largest modifiable cancer risk factor (1). By 2035, more than half the world’s population is expected to be overweight or obese, with obesity-related health expenditures reaching $1.2 trillion (2). Obesity is characterised by an excessive expansion of white adipose tissue (WAT) mediated by an increase in adipocyte number and volume (3). It is linked to an overall lower life expectancy and associated with multiple serious comorbidities, including coronary heart disease, diabetes, hypertension and cancer (4). Given the increase in the prevalence of obesity and its associated comorbidities, it is vital to understand the molecular mechanisms underpinning obesity-driven diseases. This requires models that faithfully recapitulate adipose biology and its communication with other tissues. Adipocytes are difficult to culture and maintain *in vitro* as they have poor cell–cell/cell–surface attachment, low viability and a tendency to clump together. Their large size and dynamic, high lipid content present methodological challenges, for example, when performing mass-spec analysis and single-cell RNA-Seq. Over the years, a limited number of mouse and human-derived primary pre-adipocyte cell lines have been established (5). However, primary pre-adipocytes isolated from fresh tissue are available in small amounts with limited renewal capacity and they cannot be maintained in culture over long periods (3). Thus, researchers are often required to use cell lines that are of limited relevance to their studies, particularly since different adipose depots show diversity in terms of cellular composition, proportion of white vs brown adipose tissue (BAT) and adipocyte behaviour, and are strongly influenced by their interactions with neighbouring tissues.

Adipose tissue is anatomically divided into visceral and subcutaneous adipose tissue and is functionally classified as WAT (largely for energy storage), BAT (energy expenditure as heat to regulate body temperature) and beige adipose tissue, which retains characteristics of both (6). Importantly, adipose tissue from different anatomical locations can show significant heterogeneity in terms of adipocyte metabolism, insulin sensitivity, adipokine production and overall secretome and extracellular matrix production (7). The prostate, for example, is surrounded by peri-prostatic adipose tissue (PPAT), the volume of which is associated with an increased risk of lethal prostate cancer, an enhanced extra-prostatic invasion, a faster disease progression and a reduced therapy response (8, 9). Despite not being in the peritoneal cavity, PPAT is considered as visceral adipose tissue, functionally showing both WAT and BAT characteristics (beige adipose tissue) (10, 11). Unlike WAT, the volume of PPAT is independent of body mass index. It has a higher percentage of adipose-derived stem cells compared to WAT and secretes higher levels of pro-inflammatory cytokines and MMP2/9 (10, 12, 13). However, PPAT is still poorly characterised and its contribution to prostate cancer and changes driven by obesity remain unknown; therefore, new models are needed to better understand its biology.

In recent years, several methods have been developed to culture pre-adipocytes and mature adipocytes *in vitro*. These include growing pre-adipocytes or components of adipose tissue as scaffold-free spheroids or spheroids embedded in cellular matrices, including but not limited to Matrigel (14), collagen fibres (15) and hydrogel (16). While spheroid models provide cell–cell contact and allow studying adipocytes in a 3D environment, it is technically challenging to form consistent, well-defined spheroids in culture, and they fail to recapitulate the heterogeneity of adipose tissue complexity. Moreover, different cellular matrices can encourage fibroblast growth and have different effects on adipocyte behaviour (5). To maintain longer adipocyte viability, microfluidic systems were developed with improved nutrient access (5, 17). These models can better mimic natural physiology; however, they are difficult to scale up and require specialised equipment. Finally, mature adipocytes isolated from different fat depots can be grown as ceiling cultures (18) or under trans-well inserts (19). Ceiling cultures can be used to study differentiation of adipose tissue (20), and they extend cell viability. However, over time, mature adipocytes dedifferentiate in culture and they can be challenging to scale up (5).

Here, we outline a protocol for *ex vivo* culture of fresh PPAT and non-prostatic adipose tissue (NPAT), which reflects donor- and depot-specific characteristics. *Ex vivo* culture of PPAT/NPAT explants maintains complex cell–cell interactions within adipose tissue and preserves local tissue architecture. We have also described establishment of immortalised, patient PPAT-derived pre-adipocytes with patient-matched NPAT pre-adipocytes that can be *in vitro* differentiated into mature adipocytes bearing characteristic markers. Although we focus here on PPAT, these protocols could be readily adapted to other ‘organ-associated’ fat depots, such as mammary adipose tissue, and to tissues of non-human origin.

### Ethics statement

Clinical samples and patient data were collected and processed following approval from the London South East NHS Research Ethics Committee (REC) and Health Research Authority (HRA) (IRAS number 262452). Informed consent was obtained from all participants, ensuring their full understanding of the study procedures. Participant data were collected and stored confidentially, with all identifying information anonymised to protect privacy.

## Explant culture of fresh patient-derived peri-prostatic adipose tissue

### Materials

#### Equipment

1. Cell culture hood.

2. Tissue culture incubator: 37°C, 5–10% (v/v) humidified CO_2_ incubator.

3. Laboratory scales.

4. Sterile forceps and scissors.

#### Reagents and plasticware

1. Explant culture media: Medium 199 (Sigma-Aldrich, UK; cat no: M4530-500ML); 0.01 mg/mL human insulin (Sigma-Aldrich, Germany; cat no: I9278-5ML); 5 mg/mL hydrocortisone (Tokyo Chemical Industry, Belgium; cat no: H0533); 0.01 mg/mL gentamicin (Sigma-Aldrich, USA; cat no: G1397-10ML); 1% L-glutamine (Gibco Life Technologies, UK; cat no: G7513-100ML).

2. HBSS 1× (Gibco Life Technologies, UK; cat no: 14025-050).

3. RPMI (Sigma-Aldrich, UK; cat no: R5886-500ML).

4. 70% ethanol and 10% Chemgene for sterilisation of surfaces/materials.

5. Sterile 10 cm plates (Corning, USA; cat no: 430167).

### Methods

1. Patient PPAT and NPAT samples are transported and kept in cold RPMI on ice until they are processed. Ideally, samples should be processed as soon as possible to minimise tissue degradation. Typically, the time between tissue harvesting and laboratory processing is 1.5 h, owing to the need to transfer material between different hospital sites.

2. Using forceps, transfer PPAT/NPAT samples into a 10 cm plate with cold HBSS, gently immerse and wash to remove blood, any burnt diathermy parts and blood clots, until HBSS runs clear. HBSS may need to be changed several times.

3. Once clean, blot dry and weigh total tissue and return to cold HBSS.

4. Divide samples into 1–2 mm pieces at approximately 0.5 g per 10 cm plate in 15 mL explant media and place them in the incubator. A minimum starting mass of 1 g NPAT/PPAT is recommended; however, explant cultures can be set up in smaller plates if tissue quantities are limiting.

5. Discard media after 24 h and replace with fresh 15 mL explant media as the early medium likely contains inflammatory factors associated with tissue damage from surgery.

### Results

**Figure 1:**
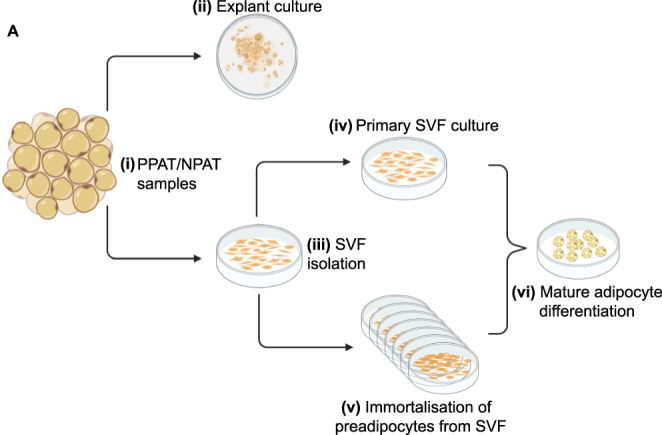
Schematic overview of culturing fresh patient peri-prostatic adipose tissue and matched NPAT, isolation of SVF, *in vitro* differentiation of mature adipocytes and generation of novel immortalised pre-adipocyte cell lines. (i) PPAT and matched NPAT from robotic-assisted radical prostatectomy can be cultured (ii) *ex vivo* as explants for at least 96 h. (iii) Alternatively, cell-specific effects of mature adipocytes can be studied by isolating SVF and either (iv) culturing primary pre-adipocytes or (v) immortalising primary pre-adipocytes to generate patient-derived cell lines that represent donor- and depot-specific characteristics. (vi) Both primary and immortalised pre-adipocytes can be *in vitro* differentiated into mature adipocytes by adding adipocyte differentiation media. This figure was created using BioRender.com.

**Figure 2:**
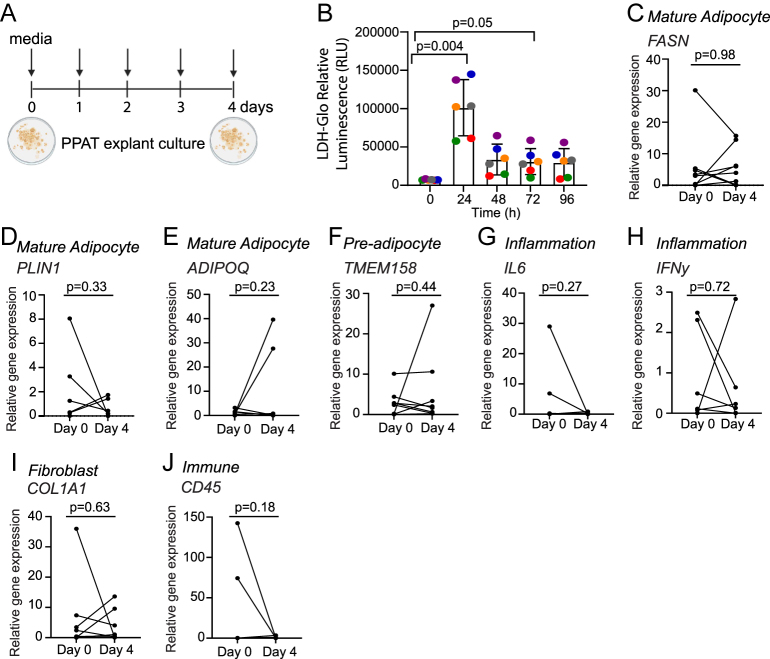
PPAT is viable and retains mature adipocyte characteristics in explant culture. (A) PPAT viability and expression of mature adipocyte markers were determined by collecting media every day from explant cultures between days 0 and 4 and sampling PPAT on days 0 and 4, respectively. This figure was created using BioRender.com. (B) Viability of PPAT explants at 0–96 h. LDH-Glo cytotoxicity assay (Promega, USA) was used according to the manufacturer’s instructions to test the viability of explant cultures. Samples were taken on days 0–96 h and diluted 1:100 in an assay storage buffer. The average expression of 6 patient samples with 2 technical repeats per patient is shown. The error bars represent ± SD. One-way ANOVA was used for statistical analysis. Individual, colour-coded points represent different patient samples. (C, D, E, F, G, H, I, J) qRT-PCR analysis of mature adipocyte markers (C) *FASN*, (D) *PLIN1* and (E) *ADIPOQ*; pre-adipocyte marker (F) *TMEM158*; inflammatory markers (G) *IL-6* and (H) *IFN-γ*; fibroblast marker (I) *COL1A1*; and immune cell marker (J) *CD45* on day 0 and day 4 of primary PPAT explant culture. Note: a reduction in inflammatory markers is expected between days 0 and 4, consistent with observations in previous studies. Data from 7 to 8 patient samples with 3 technical repeats per patient were normalised to the average of *L19*, *β-ACTIN* and *5s rRNA*. The error bars represent ± SD. Two-tailed Student’s *t*-test was used for statistical analysis. RNA from explant tissues was extracted following the instructions of Monarch Total RNA Miniprep kit.

Patient-derived PPAT/NPAT samples collected from robotic-assisted radical prostatectomy can be cultured *ex vivo* for at least 96 h (Fig. 1) with no significant changes in cell viability after 24 h (Fig. 2A and B). Patient-matched NPAT/PPAT samples were collected from the same procedure. NPAT was sampled from the pre-peritoneal fat, which is located between visceral and subcutaneous fat tissue. There were also no significant changes in *FASN*, *PLIN1* and *ADIPOQ* (mature adipocytes); *TMEM158* (pre-adipocyte); *IL-6* and *IFN-γ* (inflammatory markers); *COL1A1* (fibroblast marker); and *CD45* (immune cell marker) levels (Fig. 2C, D, E, F, G, H, I, J). The variability in these markers represents inter-patient heterogeneity in terms of numbers and types of immune cells and other cell types present in adipose tissue reinforcing the importance of being able to isolate and experimentally manipulate pure adipocyte populations. There was an expected reduction in inflammatory markers between days 0 and 4, consistent with observations in previous studies (21, 22). Pro-inflammatory markers can increase after surgical resection due to tissue damage and a natural response to surgical trauma; however, they decrease in culture within 24–48 h (21). Once established, adipokines or other secreted proteins can be isolated from explant conditioned media for further experiments. Explant cultures can also be used for co-culture experiments or stained for H&E and immunohistochemistry staining.

## Isolation and *in vitro* differentiation of peri-prostatic adipose tissue stromal vascular fraction (SVF)

Adipose tissue is comprised of mature adipocytes and a heterogeneous population of cells, including mesenchymal stem cells, pre-adipocytes, fibroblasts, immune cells, endothelial progenitor cells and red blood cells, collectively known as the SVF (21). Mesenchymal stem cells isolated from the SVF of PPAT/NPAT can be *in vitro* differentiated into mature adipocytes (Fig. 1).

The steps outlined below follow a typical enzymatic SVF isolation protocol (22, 23, 24), but with enzymes and digestion time optimised for PPAT/NPAT samples to minimise cell death and maximise tissue dissociation.

### Materials

#### Equipment

1. Cell culture hood.

2. Tissue culture incubator: 37°C, 5–10% (v/v) humidified CO_2_ incubator.

3. Lab scale.

4. Forceps and scissors.

5. Shaking incubator (for example, GFL shaking incubator 3031).

#### Reagents and plasticware

1. Enzyme digestion mix: DMEM/F12 (Gibco Life Technologies, USA; cat no: 11320-033); 3% BSA (Sigma-Aldrich, USA; cat no: A3803-100G); 1 mg/mL collagenase type I (Sigma-Aldrich, USA; cat no: C0130-500MG); 1 mg/mL collagenase type II (Sigma-Aldrich, Israel; cat no: C6885-500MG); 2 mg/mL Dispase II (Sigma-Aldrich, USA; cat no: D4693-1G); 20 units/mL DNase (added immediately before use) (New England Biolabs; cat no: M0303L).

2. Basal media: DMEM/F12; 17 μM pantothenic acid (Sigma-Aldrich, USA; cat no: 21210-5G-F); 33 μM biotin (Sigma-Aldrich, USA; cat no: B4501-1G); 1% penicillin–streptomycin–glutamine (Gibco Life Technologies, USA; cat no: 10378016).

3. Inoculation media: basal media; 10% FCS.

4. Proliferation media: basal media; 10 ng/mL EGF (Gibco Life Technologies, USA; cat no: PHG0315); 1 ng/mL FGF (Gibco Life Technologies, USA; cat no: PHG0024); 2.5% FCS.

5. Differentiation media: basal media; 0.07 μM human insulin (Sigma-Aldrich, Germany; cat no: I9278-5ML); 1 nM triiodothyronine (T3) (Sigma-Aldrich, USA; cat no: T6397-100MG); 500 μM 3-isobutyl-1-methylxanthine (IBMX) (Sigma-Aldrich, USA; cat no: 15879-250MG); 0.03 μM dexamethasone (Sigma-Aldrich, USA; cat no: D1756-100MG); 0.2 mM indomethacin (Sigma-Aldrich, USA; cat no: 17378-5UG).

6. Maintenance media: basal media; 0.07 μM human insulin; 1 nM T3; 0.03 μM dexamethasone.

7. HBSS 1x (Gibco Life Technologies, UK; cat no: 14025092).

8. 70% ethanol and 10% Chemgene for sterilisation of surfaces/materials.

9. 100 μM nylon mesh strainer (Fisherbrand; cat no: 22363549) and syringes.

10. 0.2 μm filter (Sartorius, Germany; cat no: 16534-K).

11. 12-well (Corning, USA; cat no: 3513) and 6-well (Corning, USA; cat no: 3516) plates.

### Methods

1. Using sterile forceps, gently blot dry adipose tissue on tissue paper and place immediately in cold DMEM/F12. Retain on ice if processing more than one sample.

2. Weigh samples by placing on a sterile 10 cm dish on the laboratory balance and place back in cold DMEM/F12 until further processing.

3. Prepare fresh enzyme digestion mix and filter sterilise through a 0.2 μm filter. The ratio of adipose tissue to digestion mix should be 1:2 (g:mL).

4. Rinse patient adipose tissue samples twice with HBSS, or until HBSS looks clear.

5. Place adipose tissue on a sterile 10 cm dish and mince to a fine paste using scissors, leaving pieces no larger than 1 mm in diameter. Tissue can be periodically spread with scissors to check uniformity of mincing.

6. Transfer minced tissue into a 50 mL Falcon tube containing the enzyme digestion mix using forceps.

7. Place the Falcon tubes with minced tissue in a shaking incubator at 37°C, 200 rpm for 30 min or until complete digestion. Fully digested tissue should appear thicker, and there should be no visible chunks of undigested tissue left.

8. Add 5 mL of inoculation media to neutralise the enzyme digestion mix and filter through a 100 μm strainer into a fresh tube. Centrifuge for 5 min at 270 ***g*** at room temperature. Gently aspirate the supernatant-containing mature adipocytes.

9. Resuspend the remaining pellet (SVF) containing vascular, stromal and immune cells and adipocyte progenitors in 1–2 mL of inoculation media depending on the amount of tissue, and plate in 12- or 6-well plates.

10. Replace inoculation media with proliferation media after 24 h and continue replacing media every 2–3 days until cells reach 80–90% confluency. 2–3 grams of starting adipose tissue plated per well of a 6-well plate should reach 80–90% confluency in about a week. Cells may need washing with HBSS prior to changing media to remove any remaining red blood cells and immune cells from the SVF.

11. Once pre-adipocytes reach 80–90% confluency, they can be differentiated into mature adipocytes by changing media to adipocyte differentiation media. The differentiation process takes 3–5 days, after which lipid droplets are visible under a bright-field microscope. After 5 days, mature adipocytes can be kept in maintenance media, replacing media every 3–4 days.

12. Perform qPCR and western blotting (as described in (25)) to confirm expression of adipocyte markers, and Oil Red O staining (Supplementary Materials (see section on Supplementary materials given at the end of the article)) to visualise lipid droplets. Supplementary Tables 1 and 2 list primers and antibodies used for mature adipocyte characterization.

### Results

**Figure 3:**
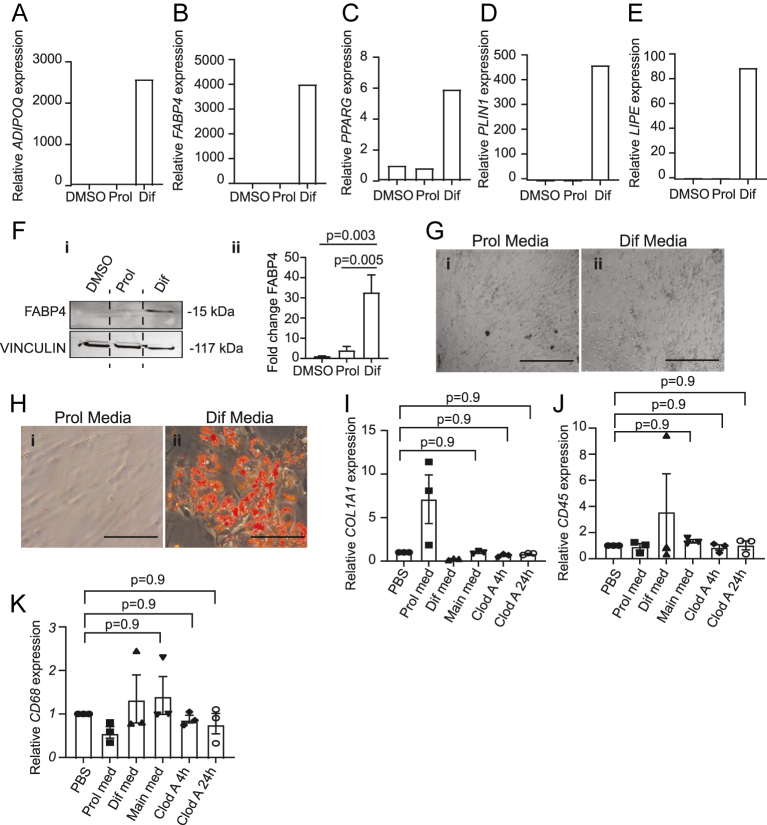
Mature adipocytes *in vitro* differentiated from PPAT pre-adipocytes express characteristic adipocyte markers and form lipid droplets. qRT-PCR analysis of mature adipocyte markers (A) *ADIPOQ*, (B) *FABP4*, (C) *PPARG*, (D) *PLIN1* and (E) *LIPE* in mature adipocytes *in vitro* differentiated from patient PPAT SVF, showing highest expression in adipocyte differentiation (Dif) media compared to DMSO and proliferation (Prol) media. Representative results of 1 out of 3 patient-specific graphs with 3 technical repeats per patient are shown due to the high variability observed between patient samples. Patient-specific graphs of the remaining repeats can be found in Supplementary Fig. 1A, B, C, D, E, F, G, H, I, J. Data normalised to the average of *GAPDH* and *β-ACTIN*. (F) (i) Western blot analysis of FABP4 (mature adipocyte marker) in mature adipocytes *in vitro* differentiated from patient PPAT SVF cultured in adipocyte differentiation media compared to DMSO and proliferation media. Representative results of 1 out of 3 patient samples are shown. VINCULIN was used as a housekeeping protein and loading control. (ii) Quantification of western blot results (i) normalised to housekeeping control showing average of 3 patient samples is presented. The error bars represent ± SD. Two-tailed Student’s *t*-test was used for statistical analysis. (G and H) Representative bright-field microscopy images showing (G) acquisition of mature adipocyte morphology and (H) lipid droplet formation (assessed by Oil Red O staining) *in vitro* after switching SVF cultures from (i) proliferation medium to (ii) differentiation media. Scale bar: 400 μm. (I, J, K) qRT-PCR analysis of (I) fibroblast (*COL1A1*), (J) general immune (*CD45*) and (K) macrophage (*CD68*) levels in pre-adipocytes kept in proliferation (Prol), differentiation (Dif) and maintenance (main) media. Fibroblast and immune marker levels remained extremely low in maintenance media. 4 and 24 h clodronate liposome depletion of macrophages did not significantly decrease immune marker levels, suggesting very low levels of immune cell infiltration. Data normalised to PBS control and the average of *GAPDH* and *β-ACTIN*. Combined results of 3 patients with 3 technical repeats per patient are shown. The error bars represent ± SD. One-way ANOVA was used for statistical analysis. Pre-adipocytes were kept in proliferation media until 80–90% confluency and then changed to differentiation media for 5 days. After 5 days in differentiation media, mature adipocytes were kept in maintenance media.

*In vitro* differentiated mature adipocytes expressed mature adipocyte markers at the RNA (Fig. 3A, B, C, D, E) and protein level (Fig. 3F(i-ii)). Expression levels of mature adipocyte markers varied across patient samples; however, they all showed the same trend of increased levels in differentiation media compared to proliferation media, indicating successful differentiation (Fig. 3A, B, C, D, E, Supplementary Fig. 1A, B, C, D, E, F, G, H, I, J). Within 3–5 days of adding adipocyte differentiation media, there was a significant change in cell morphology from spindle-shaped cells of fibroblast-like morphology (Fig. 3G(i)), to rounder, mature adipocyte-like appearance (Fig. 3G(ii)), and evident lipid droplet formation as indicated by Oil Red O staining (Fig. 3H). Mature adipocytes kept in adipocyte differentiation and maintenance media had minimal fibroblast (Fig. 3I) and immune cell contamination (Fig. 3J and K). Clodronate liposome depletion of macrophages in differentiated mature adipocytes kept in maintenance media did not significantly decrease *CD45* and *CD68* levels, suggesting the presence of low numbers (Fig. 3J and K).

## Immortalisation of pre-adipocytes isolated from the stromal vascular fraction of peri-prostatic adipose tissue

**Figure 4:**
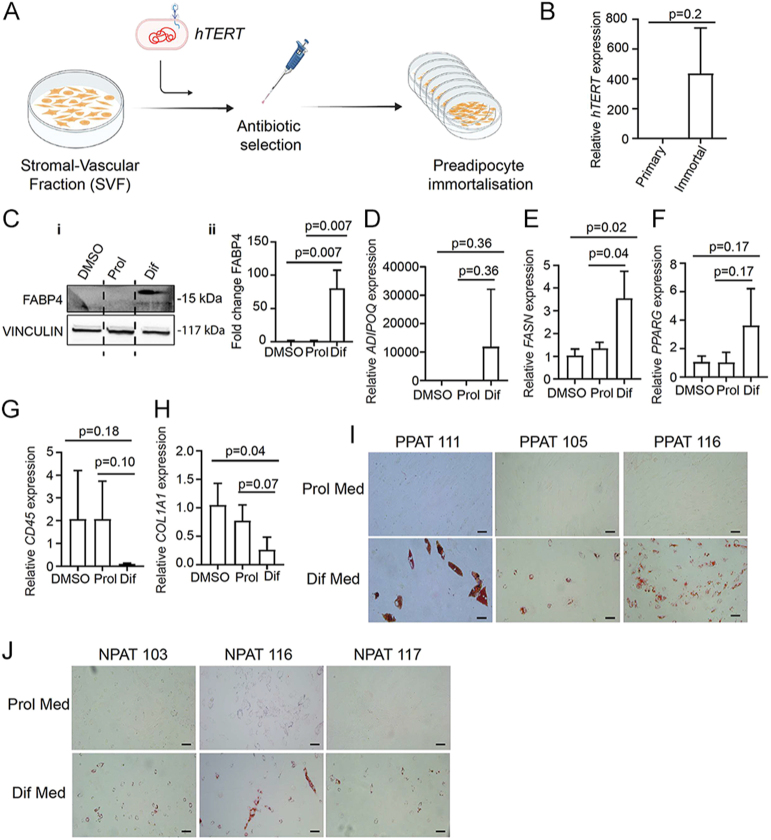
Pre-adipocytes isolated from human PPAT immortalised and differentiated *in vitro* express mature adipocyte markers and very low levels of fibroblast and immune cell contamination. (A) Schematic diagram summarising PPAT SVF pre-adipocyte immortalisation. PPAT SVF is kept in proliferation media until pre-adipocytes reach 80–90% confluency. Cells are then immortalised by lentiviral delivery of human telomerase reverse transcriptase (hTERT) followed by appropriate antibiotic selection for 2 weeks. Following immortalisation, pre-adipocytes can be cultured routinely and differentiated into mature adipocytes. This figure was created using BioRender.com. (B) qRT-PCR analysis showing *hTERT* expression in primary vs immortalised pre-adipocytes. Although there was significant patient variability, *hTERT* expression levels were elevated in immortalised pre-adipocytes compared to primary pre-adipocytes. The average of 4 patient samples (3 technical repeats per patient) is shown. The error bars represent ± SD. Data normalised to *GAPDH*. Two-tailed Student’s *t*-test was used for statistical analysis. (C) (i) Western blot results showing higher protein levels of FABP4 (mature adipocyte marker) in immortalised PPAT SVF pre-adipocytes cultured in adipocyte differentiation media compared to DMSO and proliferation media. Representative results of 1 out of 3 patients are shown. VINCULIN was used as a housekeeping protein and loading control. (ii) Quantification of western blot results (i) normalised to housekeeping control showing average of 3 patient samples is presented. The error bars represent ± SD. Two-tailed Student’s *t*-test was used for statistical analysis. (D, E, F, G, H) qRT-PCR analysis of mature adipocyte markers (D) *ADIPOQ*, (E) *FASN* and (F) *PPARG* showing highest expression in adipocyte differentiation (Dif) media compared to DMSO and proliferation (Prol) media. (G) General immune marker (*CD45*) and (H) fibroblast marker (*COL1A1*) showing low levels in differentiation media compared to DMSO and proliferation media, suggesting low levels of contamination. Immortalised patient pre-adipocytes were kept in proliferation media until 80–90% confluency and then changed to differentiation media for 10 days. The average of 3 patient samples (3 technical repeats per patient) is shown. The error bars represent ± SD. Data normalised to *GAPDH* and DMSO control. Two-tailed Student’s *t*-test was used for statistical analysis. (I and J) Bright-field microscopy images showing lipid droplet formation in (I) PPAT and (J) NPAT stained with Oil Red O. Lipid droplet formation was observed when immortalised pre-adipocytes were changed from proliferation media to differentiation media. Scale bar: 25 μm. Note that lipid droplet formation takes longer in immortalised pre-adipocytes (10 days) compared to primary cells (3–5 days). Pre-adipocytes were cultured in differentiation media for 10 days.

Primary pre-adipocytes isolated from patient PPAT/NPAT can be immortalised with human telomerase reverse transcriptase (hTERT) (26) (Fig. 4A).

### Materials

#### Equipment

1. Cell culture hood.

2. Tissue culture incubator: 37°C, 5–10% (v/v) humidified CO_2_ incubator.

#### Reagents and plasticware

1. jetPRIME transfection reagent (Polyplus-Sartorius, Germany; cat no: 101000046).

2. Polybrene (Millipore, USA; cat no: TR-1003-G).

3. ViralBoost Reagent (Alstembio, USA; cat no: VB100).

4. *pCWX-UBI-hTert-PGK-BSD* (12,278 bp) from Addgene (#114316).

5. Lentiviral packaging plasmids: pCMV-dR8.91 (12,150 bp) and pMD2-G (5,824 bp).

6. Blasticidin (MedChem Express, USA; cat no: HY-K1054-1ML).

7. 70% ethanol and 10% Chemgene for sterilisation of surfaces/materials.

8. 0.45 μm PES filter (Sartorius, Germany; cat no: 16537).

9. 10 cm plates (Corning, USA; cat no: 430167) and 6-well plates.

### Methods

#### Generation of pCWX-UBI-hTERT-PGK-BSD lentiviral vectors

1. Plate six 10 cm plates (can scale down, if desired) of 2 × 10^6^ HEK293T cells per plate 24 h prior to transfection.

2. Transfect cells with hTERT plasmid and lentiviral packaging plasmids (4:1:4 molar ratio, pCMV-dR8.91:pMD2-G:*pCWX-UBI-hTert-PGK-BSD*, total 10 μg per plate) using jetPRIME transfection reagent according to the manufacturer’s instructions (alternative transfection reagents may be used).

3. 4 h post-transfection, aspirate the cell culture medium and add 10 mL fresh medium containing ViralBoost Reagent (1:500 dilution) and allow viral production for further 72 h.

4. Pool together media containing viral particles, centrifuge at 0.4 g for 5 min to remove cells, and filter through a 0.45 μm filter. Use immediately or prepare 1 mL aliquots for storage at −80°C.

#### Transduction of pre-adipocytes with hTERT lentiviral vectors

5. Add 1 mL of virus and polybrene to a final concentration of 4 μg/mL to primary PPAT/NPAT pre-adipocytes at 80–90% confluency (1 mL of virus to 2 mL of proliferation media per well/6-well plate).

6. Change medium after 24 h and allow infection for a further 72 h.

7. Start antibiotic selection by adding Blasticidin at 5 μg/mL and changing medium every other day for one week. Add Blasticidin at 10 μg/mL and change medium every other day for another week. Note that Blasticidin concentration may require optimisation for pre-adipocytes from different depots.

#### Growth and maintenance of immortalised human peri-prostatic pre-adipocytes

9. Once all non-transduced cells are dead, let immortalised human PPAT/NPAT pre-adipocytes grow without antibiotic in proliferation media at 37°C in a humidified incubator with 5% CO_2_.

10. When immortalised pre-adipocytes reach 80–90% confluency, they can be split and vials can be frozen (freezing media: proliferation media with 5% DMSO). Note: ensure that cultures are free from mycoplasma contamination and expression of pre-adipocyte markers is confirmed.

11. For differentiation into mature adipocytes, place immortalised pre-adipocytes in differentiation media for 10 days, after which they can be transferred to maintenance media.

12. Confirm differentiation to mature adipocytes by qPCR and western blotting of the above-described markers and by Oil Red O staining of lipid droplets (Supplementary Materials). Note that lipid droplet formation takes longer in immortalised pre-adipocytes (10 days) compared to primary cells (3–5 days).

### Results

**Figure 5:**
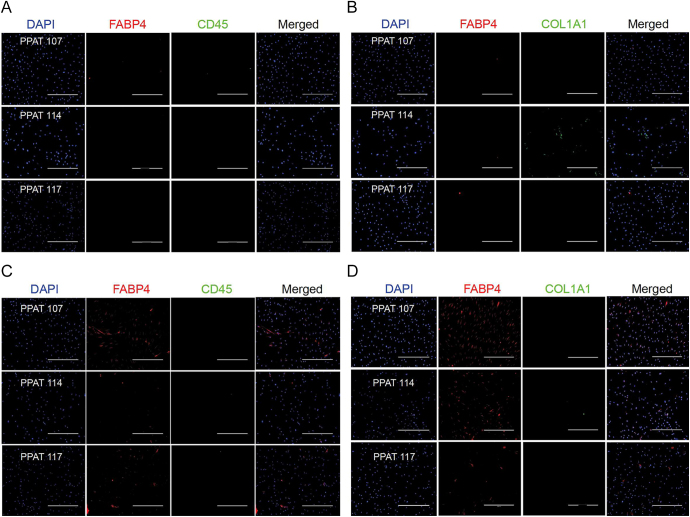
Immortalised PPAT pre-adipocytes differentiate into mature adipocytes with minimal fibroblast and immune cell contamination. (A, B, C, D) Immortalised peri-prostatic (A and B) pre-adipocytes and (C and D) mature adipocytes stained with FABP4 (mature adipocyte), CD45 (immune), COL1A1 (fibroblast) and DAPI (nuclear marker). Pre-adipocytes in proliferation media were cultured on coverslips until they reached 80–90% confluency and then fixed with 1% formalin for an hour at room temperature and stained with antibodies for FABP4, CD45 and COL1A1. Immunofluorescence was performed as described in (31). Mature adipocytes were kept in differentiation media for 10 days before fixing. All images were taken using an EVOS cell imaging system. Exposure time was the same across different samples for each marker. Refer to Supplementary Table 2 for a list of antibodies used. Scale bar: 400 μm.

**Figure 6:**
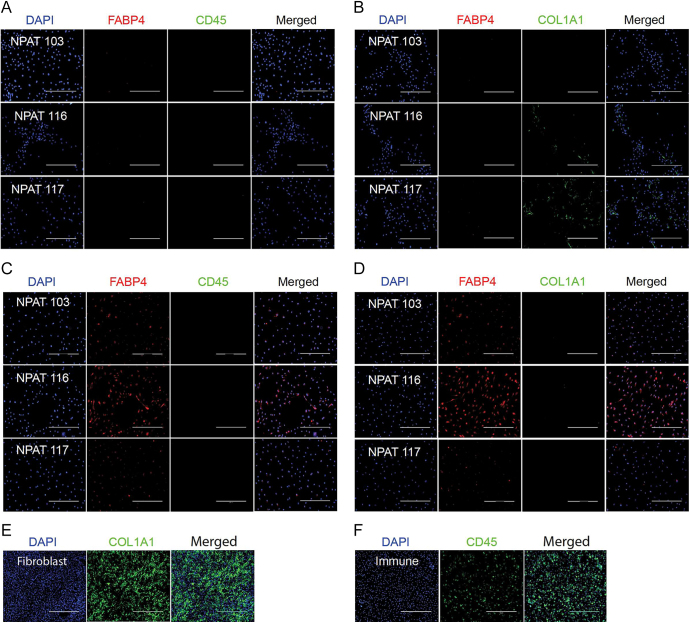
Immortalised NPAT pre-adipocytes differentiate into mature adipocytes with minimal fibroblast and immune cell contamination. (A, B, C, D) Immortalised non-prostatic (A and B) pre-adipocytes and (C and D) mature adipocytes stained with FABP4 (mature adipocyte), CD45 (immune), COL1A1 (fibroblast) and DAPI (nuclear marker). Pre-adipocytes in proliferation media were cultured on coverslips until they reached 80–90% confluency, then fixed and stained. Mature adipocytes were kept in differentiation media for 10 days before fixing. Images were taken using an EVOS cell imaging system. (E) Fibroblasts stained with COL1A1 and DAPI (nuclear marker). Fibroblasts were grown on coverslips for 10 days, changing media every 2 days until they were confluent, then fixed with 1% formalin for an hour at room temperature and stained with antibodies for FABP4, CD45 and COL1A1. Scale bar: 400 μm. (F) PBMC-derived human macrophages stained with CD45 and DAPI (nuclear marker). Fully differentiated macrophages were grown on cover slips, fixed and stained. All images were taken using an EVOS cell imaging system. Immunofluorescence was performed as described in (31). Exposure time was the same across different samples for each marker. Refer to Supplementary Table 2 for a list of antibodies used. Scale bar: 400 μm.

Despite significant patient variation, all immortalised pre-adipocytes expressed considerably higher levels of *hTERT* compared to primary pre-adipocytes, indicating successful immortalisation (Fig. 4B). Mature adipocytes differentiated from immortalised pre-adipocytes had higher levels of mature adipocyte markers at the protein (Fig. 4C) and RNA level in differentiation media compared to proliferation media (Fig. 4D, E, F). There was substantial variation in differentiation propensity between patient samples. However, all samples tested showed the same trend of substantially increased mature adipocyte markers in differentiation media. Moreover, immune cell (*CD45*) (Fig. 4G) and fibroblast (*COL1A1*) (Fig. 4H) markers were downregulated in differentiation media suggesting low levels of contamination. Lipid droplet formation was observed 10 days after adding adipocyte differentiation media (Fig. 4I and J). Immunofluorescence staining of immortalised PPAT pre-adipocytes was negative for FABP4 and CD45 (Fig. 5A). Low levels of COL1A1 staining were observed in one of the samples, which could also be the result of extracellular matrix produced by pre-adipocytes (Fig. 5B). Immortalised PPAT pre-adipocytes differentiated into mature adipocytes, on the other hand, expressed FABP4 and were negative for CD45 and COL1A1 staining (Fig. 5C and D) consistent with very low levels of *COL1A1* detected at the RNA level (Fig. 4H). Similarly, immortalised NPAT pre-adipocytes were negative for FABP4 and CD45 staining (Fig. 6A) but showed low levels of COL1A1 expression (Fig. 6B). When differentiated into mature adipocytes, they were negative for FABP4, CD45 and COL1A1 (Fig. 6C and D). Fibroblasts and PBMC-derived human macrophages were used as a positive control for COL1A1 (Fig. 6E) and CD45 (Fig. 6F), respectively. We have not ascertained maximum number of passages possible with the immortalised cell lines. However, we have lines that have been passaged more than twenty times while retaining capacity to divide and to differentiate into mature adipocytes *in vitro*.

## Discussion and conclusions

Here, we outline different methods of culturing patient PPAT and matching NPAT samples. *Ex vivo* culture of fresh patient PPAT samples can be maintained in culture for at least 96 h with the advantage of retaining tissue architecture and local microenvironment. Conditioned media from explants can be studied in response to various treatments to identify secreted factors/proteins of interest for further mechanistic studies. Explants can also be co-cultured with various cell types, such as immune cells, endothelial cells or cancer cells, to better understand the communication between organ-specific fat depots and their influence on the microenvironment. 3D models of PPAT or other organ-specific fat depots can be constructed by culturing explant cultures on extracellular matrix (27, 28). To focus on the cell-type-specific effects of adipocytes, SVF can be isolated from fresh patient PPAT/NPAT. Primary, fat depot-specific pre-adipocytes better represent organ and patient characteristics, such as differences in body mass index, making them useful tools in understanding adipose heterogeneity. Immortalisation of patient-derived PPAT pre-adipocytes has the added benefit of having access to proliferative, cryo-preservable cells. Continuous culture of pre-adipocytes also allows more starting material for techniques such as metabolomics/proteomics and lipidomics. Moreover, gene editing tools, such as CRISPR and siRNA-mediated gene silencing, can be potentially applied to immortalised PPAT pre-adipocytes to help shed light on adipocyte-specific mechanisms. PPAT explant cultures and patient-derived PPAT pre-adipocytes can also provide tools to better understand the bidirectional communication between PPAT and prostate cancer cells to study the complex relationship between prostate cancer progression and the tumour-adipose microenvironment (29, 30). Obesity leads to significant changes in both adipose tissue composition and its overall secretory profile (3). Obesity-mediated changes in adipose tissue can support tumour growth and development by extensive remodelling of the surrounding vasculature, changes in extracellular matrix production, particularly collagen VI, and secretion of pro-inflammatory cytokines, such as IL-6, all of which can significantly impact the prostate tumour microenvironment (29, 30). The methods we have outlined here focus on culturing patient PPAT; however, similar protocols can also be applied to other organ-specific fat depots, such as the mammary fat pad. Collectively, these tools can help us better understand adipocyte biology and help form the basis of future adipocyte targeted therapies.

## Supplementary materials







## Declaration of interest

The authors declare no conflict of interest that could be perceived as prejudicing the impartiality of the research reported.

## Funding

This work was supported by Prostate Cancer Research (grant number: 6965), the Prostate Cancer Foundation (grant number: 24YOUN18) and Prostate Cancer UKhttps://doi.org/10.13039/501100000771 (grant number: RIA23-ST2-016).
