# Supplementary figures and images for: Fat’s all, folks: culturing and manipulating peri-prostatic adipocytes to probe impacts on prostate cancer biology
Source: J Endocrinol. 2026 Jan 23;268(1):e250256. doi: 10.1530/JOE-25-0256 (PMC12849434; doi:10.1530/JOE-25-0256)

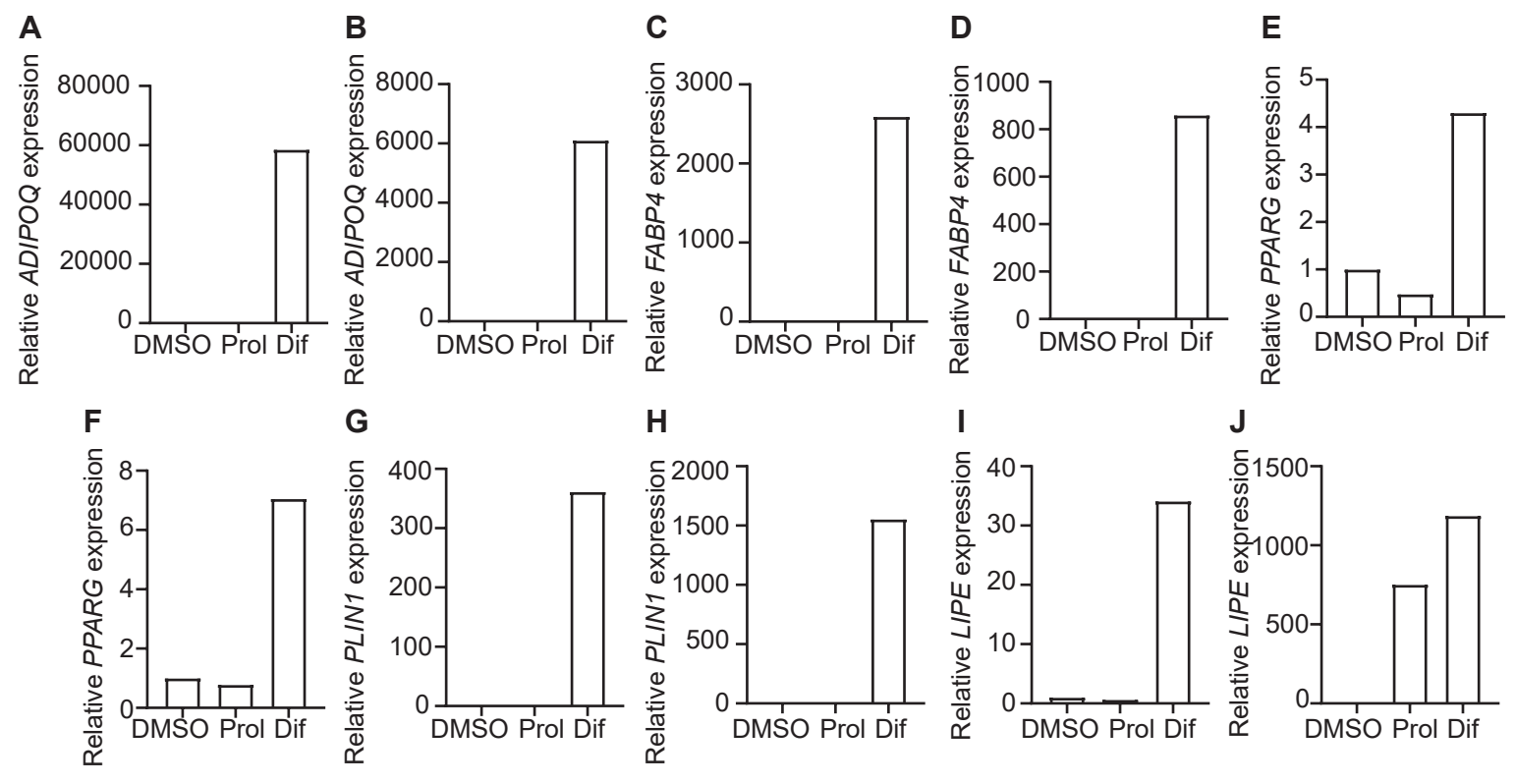

Supplement: Supplementary file 1 [file supplementary_figure_1.pdf]
