# Supplementary material for: Fat’s all, folks: culturing and manipulating peri-prostatic adipocytes to probe impacts on prostate cancer biology
Source: J Endocrinol. 2026 Jan 23;268(1):e250256. doi: 10.1530/JOE-25-0256 (PMC12849434; doi:10.1530/JOE-25-0256)
Supplement: Supplementary file 2 [file supplementary_tables.pdf]

Supplementary Table 1: List of primers used.

Gene	Forward sequence 5-3'	Reverse sequence 5-3'
GAPDH	ATGGGGAAGGTGAAGGTCG	GGGGTCATTGATGGCAACAATA
β-ACTIN	GGCATCCTCACCCTGAAGTA	GGTCATCTTCTCGCGGTTG
L19	GCGGAAGGGTACAGCCAAT	AGCAGCCGGCGCAAA
5s rRNA	TACGGCCATACCACCCTGA	GGCGGTCTCCCATCCAA
hTERT	AAATGCGGCCCCTGTTTCT	CAGTGCGTCTTGAGGAGCA
ADIPOQ	GCCTGTTTCTGACCAATC	CCACTCTCCTATTTCTGATAAC
FASN	AGTGCATCAAAGAAGCCCATC	TGACTGTGTCCTTGGAGTTGT
PLIN1	GCGGAATTTGCTGCCAACACTC	AGACTTCTGGGCTTGCTGGTGT
TMEM158	TGTGCTTCGTGCTGTAGTTATC	TCAGTCCAAGGGCTTAAACATC
FABP4	ATCAACCACCATAAAGAGAAA	AACTTCAGTCCAGGTCAA
PPARG	CGTGGATCTCTCCGTAAT	TGGATCTGTTCTTGTGAATG
LIPE	GTATGTCACGCTGCATAAGGG	CTGTCTCGTTGCGTTTGTAGT
COL1A1	AGGGCTCCAACGAGATCGAGATCCG	TACAGGAAGCAGACAGGGCCAACGTCG
CD45	CTTCAGTGGTCCCATTGTGGTG	CCACTTTGTTCTCGGCTTCCAG
CD68	CGAGCATCATTCTTTCACCAGCT	ATGAGAGGCAGCAAGATGGACC
IL6	TGCAATAACCACCCCTGACC	GTGCCCATGCTACATTTGCC
IFNγ	GGCTTTTCAGCTCTGCATCG	CGCTACATCTGAATGACCTGC

Supplementary Table 2: List of antibodies used.

Target	Species	Company	Catalog Number
β-Actin	Mouse	Abcam	ab6276
Vinculin	Mouse	Merck	V9131
Fabp4	Rabbit	Abcam	ab92501
CD45	Mouse	Cell Signalling	86532S
Col1a1	Mouse	Cell Signalling	66948S
