# Supplementary material for: Fat’s all, folks: culturing and manipulating peri-prostatic adipocytes to probe impacts on prostate cancer biology
Source: J Endocrinol. 2026 Jan 23;268(1):e250256. doi: 10.1530/JOE-25-0256 (PMC12849434; doi:10.1530/JOE-25-0256)
Supplement: Supplementary file 3 [file supplementary_materials.pdf]

Supplementary Materials and Methods

Oil Red O staining

1- Prepare a 0.35% stock of Oil Red O (Sigma-Aldrich, USA cat no: O-0625) in isopropanol, stir overnight and pass through a 0.2 µm filter. Stock solution can be kept at 4°C for up to a year.

2- Prepare Oil Red O working solution (stable for 2 h) by mixing 6 parts Oil Red O stock solution with 4 parts deionized water. Incubate at room temperature for 20 min then pass through a 0.2 µm filter.

3- Remove two thirds of medium from the well and add 4% paraformaldehyde (2.4 ml per well of a 6 well plate). Incubate plate at room temperature for 30 min.

4- Remove all paraformaldehyde and wash cells with 60% isopropanol (2.4 ml per well of a 6 well plate). Let the wells dry completely.

5- Add Oil Red O working solution (1 ml per well of a 6 well plate) and incubate the plate at room temperature for 10 min.

6- Remove all Oil Red O solution and immediately rinse under running tap water. Can take images with/out water.
